# Mitogen-activated protein kinase signal transduction and DNA repair network are involved in aluminum-induced DNA damage and adaptive response in root cells of *Allium cepa* L.

**DOI:** 10.3389/fpls.2014.00256

**Published:** 2014-06-05

**Authors:** Brahma B. Panda, V. Mohan M. Achary

**Affiliations:** Molecular Biology and Genomics Laboratory, Department of Botany, Berhampur UniversityBerhampur, India

**Keywords:** adaptive response, DNA damage, DNA repair, genome protection, MAP kinase, metabolic inhibitors, signal transduction

## Abstract

In the current study, we studied the role of signal transduction in aluminum (Al^3+^)-induced DNA damage and adaptive response in root cells of *Allium cepa* L. The root cells *in planta* were treated with Al^3+^ (800 μM) for 3 h without or with 2 h pre-treatment of inhibitors of mitogen-activated protein kinase (MAPK), and protein phosphatase. Also, root cells *in planta* were conditioned with Al^3+^ (10 μM) for 2 h and then subjected to genotoxic challenge of ethyl methane sulfonate (EMS; 5 mM) for 3 h without or with the pre-treatment of the aforementioned inhibitors as well as the inhibitors of translation, transcription, DNA replication and repair. At the end of treatments, roots cells were assayed for cell death and/or DNA damage. The results revealed that Al^3+^ (800 μM)-induced significant DNA damage and cell death. On the other hand, conditioning with low dose of Al^3+^ induced adaptive response conferring protection of root cells from genotoxic stress caused by EMS-challenge. Pre-treatment of roots cells with the chosen inhibitors prior to Al^3+^-conditioning prevented or reduced the adaptive response to EMS genotoxicity. The results of this study suggested the involvement of MAPK and DNA repair network underlying Al-induced DNA damage and adaptive response to genotoxic stress in root cells of *A. cepa*.

## Introduction

Plant genome is under constant stress from endogenous as well as exogenous factors. Plants being sedentary are uniquely equipped with innate mechanisms that help them to adapt to environmental changes. Plant genome integrity is maintained by specific cellular repair functions, among which some are known to be highly conserved during evolution (Roldan-Arjona and Ariza, 2009). DNA damage, if not repaired properly, results in genomic perturbation adversely affecting the plant development, productivity and genetic diversity (Tuteja et al., 2009; Balestrazzi et al., 2011). Recently, genetic and biochemical analysis have considerably advanced our understanding of DNA repair processes and their involvement in genotoxic stress response and signaling in plants (Ulm, 2003; Bray and West, 2005). Often, plants are known to display cross-adaptation to abiotic and genotoxic stresses (Panda et al., 2013).

The mitogen-activated protein kinase (MAPK) cascade comprising the MAPKKK-MAPKK-MAPK module is one of the major routes that mediates transduction of extra cellular stimuli to intracellular responses, which has been shown in yeast (Thevelein, 1994) and mammalian cells (Kyriakis and Avruch, 2001). MAPKs are the intracellular mediators of signals that shuttle between the cytoplasm and the nucleus targeting several transcription factors (Hirt, 2000; Yang et al., 2003a). MAPK phosphatases (MKPs) through dephosphorylation of both tyrosine and serine/threonine residues, are known to inactivate MAPK cascades with high specificity (Camps et al., 2000; Keyse, 2000). MKPs are also implicated in DNA damage response mediated by MAPK pathway (Ulm et al., 2001). Ataxia telangiectasia-mutated (ATM) and ATM-Rad3-related (ATR) kinases that are activated by DNA damage are considered central to the DNA damage response (Hurley and Bunz, 2007). DNA double strand breaks (DSBs) have been reported to activate ATM kinase, which in turn activate the downstream signaling pathways leading to transient arrest of the cell cycle and inhibition of DNA replication facilitating DNA repair (Mannuss et al., 2012). ATM is also activated by oxidative stress (Watters, 2003). On the other hand, the ATR kinase is activated by stalled replication forks, which can occur spontaneously or upon exposure to UV-irradiation or hydroxyurea (Mannuss et al., 2012). ATR regulates the slowing of the cell cycle during S phase and the G2/M progression (Abraham, 2001). In plant cells, DNA-damage activates ATM and ATR kinases via the WEE1 serine/threonine kinase transiently arresting the cell-cycle and allowing cells to repair DNA damage prior to initiation of mitosis (De Schutter et al., 2007). However, the proteins that initially sense DNA damage and initiate the signaling response are currently unknown. Poly(ADP-ribose) polymerase (PARP) and DNA-dependent protein kinase (DNA-PK) have long been proposed as DNA damage sensors. PARP is activated by DNA break, which in turn depending on the severity of the damage and amplitude of activation, can lead to either repair or programmed cell death (PCD) in cells (Briggs and Bent, 2011). PARPs and poly(ADP ribose) glycohydrolases (PARGs) are the main enzymes responsible for the posttranslational modification known as poly(ADP-ribosyl)ation implicated in DNA damage response (Briggs and Bent, 2011). These enzymes play important roles in tolerance to genotoxic stress, DNA repair, PCD, transcription, and cell cycle control in plants (Adams-Phillips et al., 2010). Current knowledge on DNA damage response emerging from plants also suggests that strand breaks trigger the DNA damage response by inducing the expression of molecular markers associated with DNA damage repair, such as the PARP, RAD51, and breast cancer (BRCA) family members (Vanderauwera et al., 2011).

The role of reactive oxygen species (ROS) in the activation of MAPK-pathways, DNA damage response, and DNA repair networks has been suggested (Varnova et al., 2002; Yang et al., 2003b). Heavy metals (Cd^2+^, Cu^2+^, Pb^2+^, Zn^2+^) are also known to activate signal transduction pathways through the ROS-mediated MAPK pathways (Jonak et al., 2004; Lin et al., 2005; Liu et al., 2010). Aluminum (Al^3+^) is a light metal that induces DNA damage through triggering the oxidative burst (Achary et al., 2012). The involvement of checkpoint regulators such as TANMEI/ALUMINUM TOLERANT2 (ALT2) and ataxia telangiectasia-mutated (ATM) and ATM-Rad3-related (ATR) kinases in the Al-dependent root growth inhibition and cell cycle arrest in *Arabidopsis thaliana* has been suggested (Rounds and Larsen, 2008; Nezames et al., 2012). Furthermore, we have recently demonstrated the role of ROS in Ca^2+^ channel signal transduction underlying Al^3+^-induced DNA damage and adaptive response (Achary et al., 2013). Also, in this study, we have shown that the Al^3+^ induces adaptive response to genotoxic stress in root cells of *A. cepa* failing to uphold the cell cycle checkpoint arrest mechanism in the underlying DNA damage and response. Furthermore, our earlier study also suggests unknown alternate pathway(s) involving the MAPK signal transduction and DNA repair network (Achary et al., 2013).

In sequel to our earlier studies (Achary and Panda, 2010; Achary et al., 2012, 2013), in the present study we investigated the involvement of MAPK signaling in DNA repair network in the Al^3+^-induced DNA damage and adaptive response to genotoxic stress in root cells of *A. cepa* L.

## Materials and methods

### Assay systems

Bulbs of onion (*Allium cepa* L., 2*n* = 16) were used as the test system. Bulbs were procured from the local farmers. Hand-picked bulbs of uniform size were scrapped so that the apices of the root primordial were exposed and their dry scales peeled off. Bulbs were then surface sterilized by rinsing in 1% sodium hypochlorite solution followed by 70% ethanol and set for rooting in sterilized moist sand in dark. After 2 days, bulbs with 2–3 cm long roots were washed in running tap water for 5–10 min and then subjected to the chosen treatments. The experiments were conducted at room temperature 25 ± 1°C in dark.

### Test chemicals and experimental solutions

The major chemicals used in the current experiments include: Aluminum chloride (AlCl_3_, HiMedia, India), ethyl methanesulfonate (EMS, HiMedia, Mumbai), and actinomycin D (ACD), 3-aminobenzamide (3-AB), 2-aminopurine (2-AP), aphidicolin (APH), caffeine (CAF), cycloheximide (CHX), cantharidin (CAN, 2,3-dimethyl-7-oxabicyclo[2.2.1] heptane-2,3-dicarboxylic anhydride), endothall, (ENT, 7-oxabicyclo[2.2.1] heptane-2, 3-dicarboxylic acid), LY-294002 (LY), PD-98059 (PD), and sodium orthovanadate (SOV), were all procured from Sigma-Aldrich, USA. Stock solutions of the chemicals were prepared in distilled water. Chemicals that were not easily dissolved in water were first dissolved in a small volume of dimethyl sulfoxide (DMSO) and then diluted with distilled water. Experimental solution of AlCl_3_ was adjusted to pH 4.5 rendering the metal in the soluble form (Al^3+^) available for plant-uptake (Kochian et al., 2004).

### Experimental design and treatment protocol

Treatments were carried out by placing the onion bulbs on 30 mL glass test tubes (Borosil®, Mumbai) with roots dipped in the experimental solutions. Depending on the specific objectives, experiments were conducted following two different treatment protocols as described as below.

#### Experiment I: effect of kinase and phosphatase inhibitors on Al^3+^-induced cell death and DNA damage in root cells of A. cepa L.

**Figure 1:**
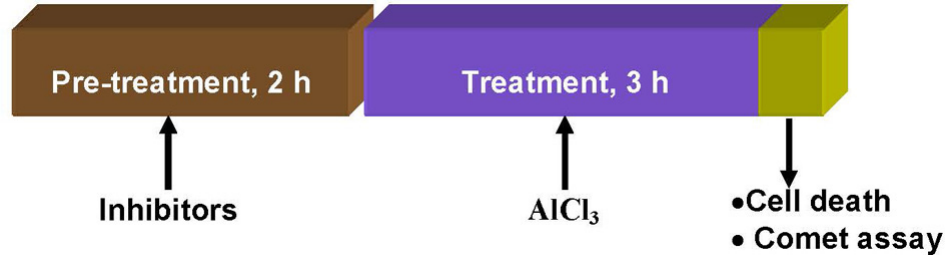
**Treatment protocol showing time and sequence of pre-treatment, treatments, and endpoints measured in root cells of *A. cepa***.

Bulbs of *A. cepa* with growing roots (2–3 cm long) were treated with Al^3+^ (800 μM) at pH 4.5 for 3 h either without or with prior treatment with the kinase inhibitors: LY(1–4 μM), PD (2.5–7.5 μM), and 2-AP (5–20 μM); protein phosphatase inhibitors: SOV (10–50 μM), ENT (10–50 μM), and CAN (5–20 μM) for 2 h. Appropriate water and DMSO controls were maintained under identical conditions for comparison. At the end of the treatments, cell death and DNA damage by the Comet assay were assayed in the excised roots (Figure 1).

#### Experiment II: effect of kinase, phosphatase, and metabolic inhibitors on Al^3+^-induced adaptive response to genotoxic stress in root cells of A. cepa L.

**Figure 2:**
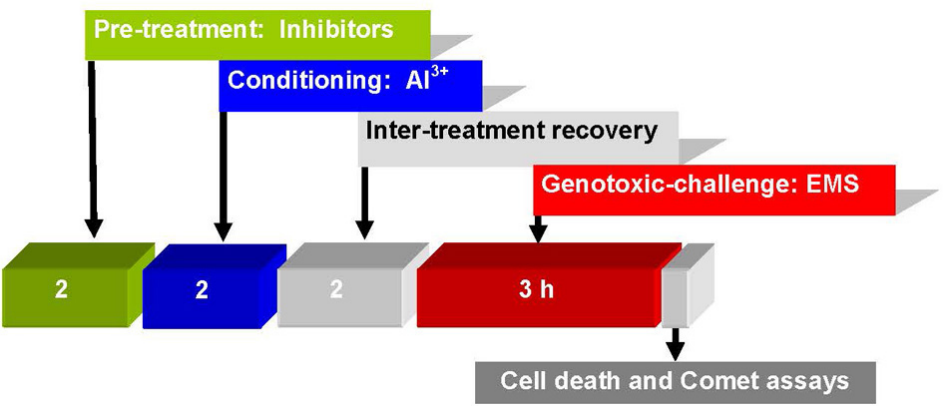
**Treatment protocol showing time and sequence of pre-treatment, conditioning, challenging treatments, cell death, and Comet assays in root cells of *A. cepa***.

Bulbs of *A. cepa* with growing roots (2–3 cm long) were first conditioned by a 2 h treatment with Al^3+^ 10 μM (pH 4.5), and after a 2 h inter-treatment interval, were subjected to challenge-treatment for 3 h by EMS 5 mM without or with pre-treatments of kinase inhibitors: LY (1, 2 μM), PD (2.5, 5 μM), 2-AP (10, 20 μM); protein phosphatase inhibitors: SOV (25, 50 μM); ENT (25, 50 μM), CAN (10, 20 μM); *de novo* translation inhibitor: CHX (1, 5 μM); transcriptional inhibitor ACD (5, 10 μM); PARP inhibitor: 3-AB (5, 10 μM), post-transcriptional repair inhibitor: CAF (10, 20 μM); repair replication polymerase inhibitor: APH (5, 10 μM) for 2 h, administered prior to Al^3+^ conditioning at low non-toxic treatments. All the treatments were terminated by washing of the intact roots in running tap water for at least 10 min. At the end of treatments, batches of roots from different groups were processed immediately for Comet assay (Figure 2). Appropriate negative (water and DMSO) and positive (inhibitor + EMS and inhibitor + low dose Al^3+^) controls were maintained and handled alike. The above pre-treatment concentrations of kinase, phosphatase, and metabolic inhibitors were chosen on the basis of pilot experiments that revealed little or no influence on EMS-induced DNA damage.

### Cell death

For determination of cell death, control, and treated bulbs with intact roots were stained with 0.25% (w/v) aqueous solution of Evans blue (HiMedia, Mumbai) for 15 min (Baker and Mock, 1994). After washing the roots, batches of 10 stained root tips of equal length (10 mm) were excised and soaked in 3 mL of *N, N*-dimethylformamide (Merck, Mumbai) for 1 h at room temperature. The absorbance of Evans blue released was measured spectrophotometrically at 600 nm.

### Comet assay

**Figure 3:**
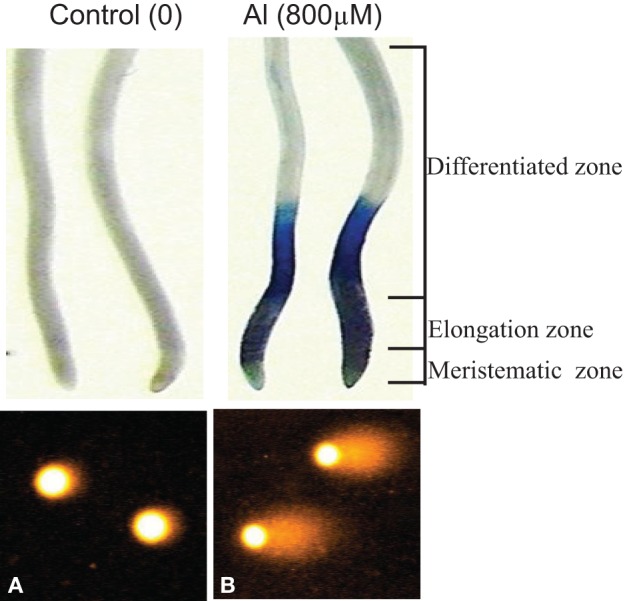
**Cell death and DNA damage in root cells of *A. cepa* visualized by Evans Blue staining and Comet assay in Control (A) and Al 800 μM (B), respectively**. Nuclei isolated from elongation and differentiation zones were analyzed for DNA damage by Comet assay.

For alkaline comet assay, bulbs with intact roots of *A. cepa* from different treatments were thoroughly washed in running tap water. Comet assay on excised roots was carried out following the protocol described earlier (Achary and Panda, 2010). Analysis of DNA damage by Comet assay was performed on the nuclei isolated from root cells belonging to the elongation or differentiation root zones (Figure 3). An Olympus BX51 microscope fitted with a fluorescence attachment (using the excitation filter 515–560 nm and barrier filter 590 nm) and a Cohu camera and Kinetic Komet™ Imaging Software 5.5 (Andor™ Technology, www.andor.com) was employed for comet analysis. Two slides prepared from 20 roots were examined for each treatment. At least, 50 comets were scored from each slide. The comet images obtained from roots of *A. cepa* were visualized and captured at 100× magnifications, respectively. Out of a number of parameters available in the software, comets were analyzed on the basis of the Olive tail moment, OTM (Kumaravel et al., 2000). The entire process of the comet assay was carried out in dim or yellow light.

### Statistical analysis

All the experiments with the exception to genotoxic assay were replicated thrice, whereas experiments with genotoxic assay were repeated at least once in order to establish the reproducibility of the results. Pooled data were statistically analyzed using analysis of variance (ANOVA), followed by Tukey's honestly significant difference (HSD) test (Daniel, 1995) employing the Windows®7/Microsoft Excel 2003 computer package.

Protection conferred by kinase and phosphatase inhibitor on Al^3+^-induced cell death and DNA-damage were calculated as percent of relative decrease from Al^3+^ (800 μM; positive control). Furthermore, adaptive response was assessed on the basis of protection calculated as percent of relative decrease of DNA damage as compared with that of the positive controls (EMS-challenge). Likewise, prevention of adaptive response induced by kinase, phosphatase and metabolic repair inhibitors was calculated as percent of the relative increase in the above values as compared to corresponding Al^3+^-conditioning plus EMS challenge (Achary and Panda, 2010).

## Results

### Effect of inhibitors of protein kinase and phosphatase on Al^3+^-induced cell death and dna damage

**Figure 4:**
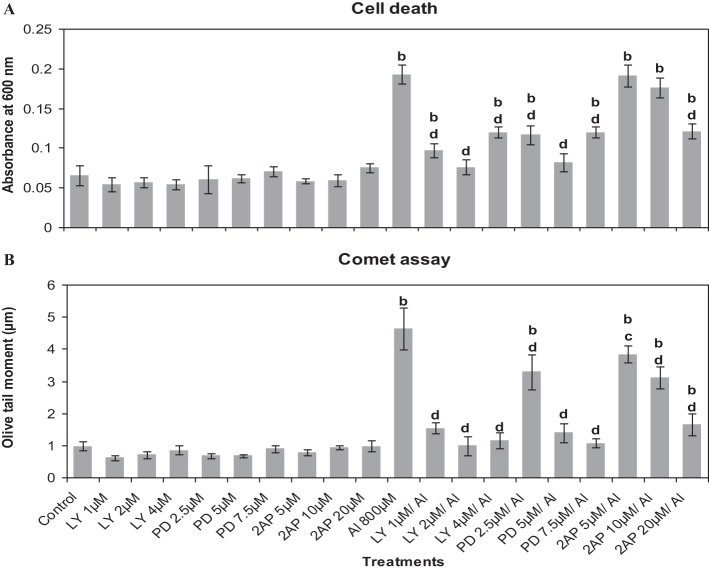
**Pre-treatment of MAPK inhibitors: LY (LY-294002) 1–4 μM; PD (PD-98059) 2.5–7.5 μM and protein kinase inhibitor: 2AP (2-aminopurine) 5–20 μM prevented Al^3+^-induced cell death (A) and DNA damage (B) in root tissue of *A. cepa***. Increase significant compared to control at *p* ≤ 0.01 (b); decrease significant compared to Al^3+^ 800 μM at *p* ≤ 0.05 (c) or 0.01 (d).

**Figure 5:**
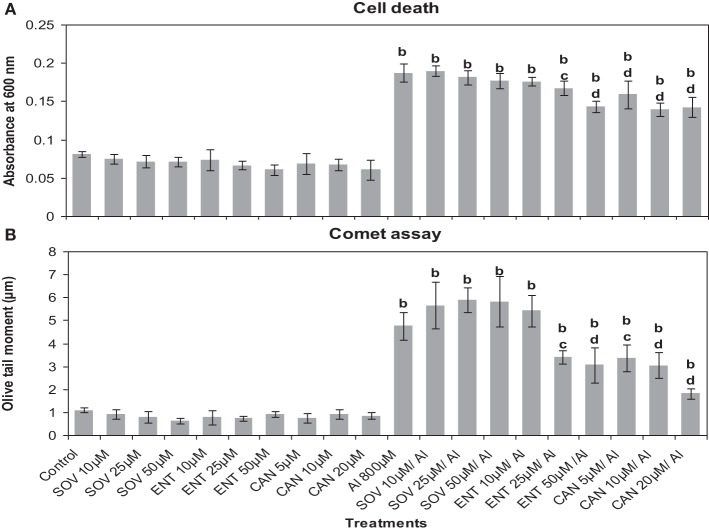
**Pre-treatment of protein phosphatase inhibitors: SOV (Na_2_VO_4_) 10–50 μM; ENT (endothall) 10–50 μM; CAN (cantharidin) 5–20 μM prevented Al-induced cell death (A) and DNA damage (B) in root tissue of *A. cepa***. Increase significant compared to control at *p* ≤ 0.01 (b); decrease significant compared to Al^3+^ 800 μM at *p* ≤ 0.05 (c) or 0.01 (d).

First of all it was established that the inhibitors at the chosen pre-treatment concentrations in par with the corresponding controls (water or DMSO) did not induce cell death or DNA damage in root cells of *A. cepa* (Figure 4). There was no difference between water and DMSO controls nor DMSO showed any effect on the treatments (data not shown). On the contrary, Al^3+^ (800 μM) induced cell death and DNA damage significantly (*p* ≤ 0.01). Al^3+^-induced cell death or DNA damage was significantly counteracted when the roots were pre-treated with the protein kinase inhibitors LY at the doses of 1, 2, and 4 μM (49.82, 60.5, and 37.7%); PD at the doses of 2.5, 5, and 7.5 μM (39.49, 57.73, and 37.7%) and 2-AP only at the dose of 20 μM (37%) that offered protection against cell death to different extents. However, 2-AP at the two lower concentrations (5 and 10 μM) failed to affect the cell death induced by Al^3+^ (800 μM). Likewise, pre-treatments with the chosen inhibitors conferred significant protection (*p* ≤ 0.05 or 0.01) against Al^3+^-induced DNA-damage in root cells (66.97, 78.61, and 75.3% inhibition by LY 1, 2, and 4 μM; 29.26, 70.14, and 77% inhibition by PD 2.5, 5, and 7.5 μM, and 17.04, 33.03, and 64.52% inhibition by 2-AP 5, 10, and 20 μM, respectively). Among the tested MAPK-inhibitors, 2-AP was the least effective in countering cell death or DNA damage induced by Al^3+^ (800 μM) (Figure 4). Similarly, pre-treatment with the protein phosphatase inhibitors (ENT 25, 50 μM and CAN 5, 10, and 20 μM) significantly protected roots from Al^3+^-induced cell death (10.6, 23.6 and 5.9, 10.6, and 23.6% protection), and DNA damage (28.3, 35.7, and 29.1, 36, and 61.9%) (Figure 5). On the other hand, SOV at doses of 10–50 μM and the ENT at the lowest dose of 10 μM apparently were ineffective in preventing Al^3+^-induced cell death or DNA damage.

### Effect of inhibitors of protein kinase and phosphatase on Al^3+^-induced genotoxic adaptation to EMS

**Figure 6:**
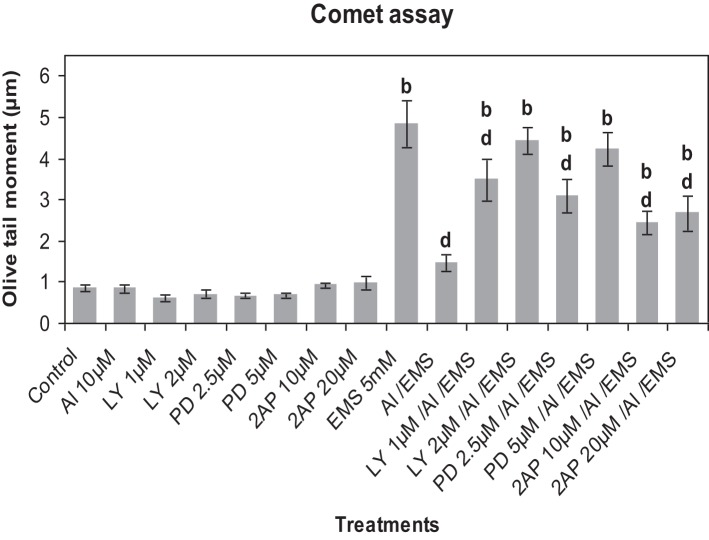
**Pre-treatments of protein kinase inhibitors: LY (LY-294002) 1 and 2 μM; PD (PD-98059) 2.5 and 5 μM; 2-AP (2-aminopurine) 10 and 20 μM, prevented Al-induced adaptive response to DNA damage induced by EMS 5 mM in root meristems of *A. cepa***. Increase significant compared to control (0) at *p* ≤ 0.01 (b); decrease significant compared to EMS challenge at *p* ≤ 0.01 (d).

**Figure 7:**
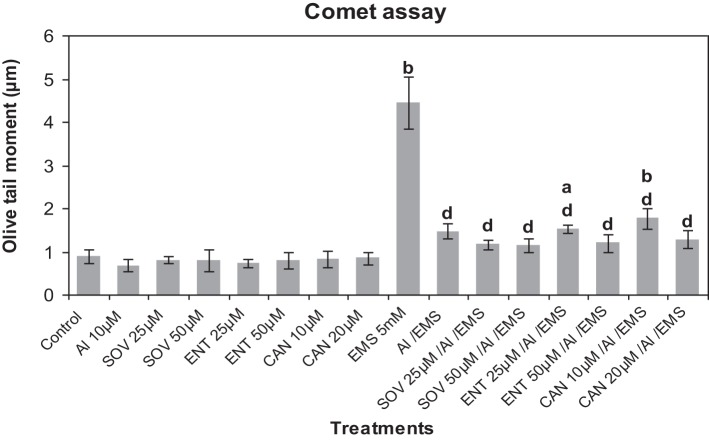
**Pre-treatments of protein phosphatase inhibitors SOV (Na_2_VO_4_) 25 and 50 μM; ENT (endothall) 25 and 50 μM; CAN (cantharidin) 10 and 20 μM prevented Al-induced adaptive response to DNA damage induced by EMS 5 mM in root meristems of *A. cepa***. Increase significant compared to control (0) at *p* ≤ 0.05 (a) or 0.01 (b); decrease significant compared to EMS-challenge at *p* ≤ 0.1 (d).

Comet analysis revealed that LY (1, 2 μM), PD (2.5, 5 μM) or 2-AP (10, 20 μM) did not significantly affect the DNA damage induced by Al^3+^ (10 μM) in root cells of *A. cepa* as compared to that in the control (Figure 6). EMS at the dose of 5 mM significantly induced (*p* ≤ 0.01) DNA damage. The kinase and phosphatase inhibitors at the tested concentrations had little or no effect on EMS-induced DNA damage (data not sown for sake of brevity). Al^3+^-conditioning significantly (*p* ≤ 0.01) protected against the EMS-induced DNA damage that accounted for 69.83% genomic protection. Pre-treatments of protein kinase inhibitors LY (1, 2 μM), PD (2.5, 5 μM) and 2-AP (10, 20 μM) significantly (*p* ≤ 0.01) abolished the Al-induced adaptive response against EMS-challenge that was evident by the recurrence of the DNA damage (58.07 and 67.1% under treatment with LY at 1, 2 μM; 52.77 and 65.45% under treatment with PD at 2.5, 5 μM; 40.06 and 45.38% under treatment with 2-AP at 10, 20 μM). Noteworthy was that of all protein kinase inhibitors LY (2 μM) and PD (5 μM) were the most effective to abolish the Al-adaptive response against EMS-challenge (Figure 6). On the contrary, the protein phosphatase inhibitors SOV (25, 50 μM), ENT (25, 50 μM), and CAN (10, 20 μM) proved ineffective to counter the Al-induced adaptive response to EMS-genotoxicity, revealed in Comet assay (Figure 7).

### Effect of metabolic inhibitors on Al^3+^-induced genotoxic adaptation to EMS

**Figure 8:**
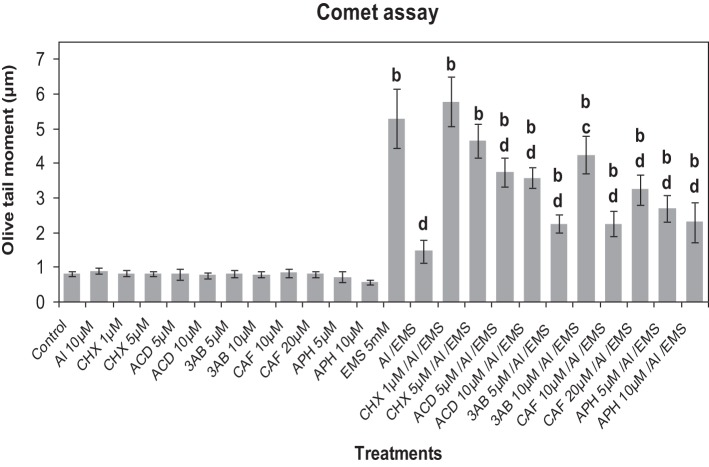
**Pre-treatments of inhibitors of protein synthesis: CHX (cycloheximide) 1 and 5 μM; transcription: ACD (actinomycin D) 5 and 10 μM; PARP: 3AB (3-aminobenzamide) 5 and 10 μM; post-transcriptional repair: CAF (caffeine) 10 and 20 μM; replication repair: APH (aphidicolin) 5 and 10 μM prevented Al-induced adaptive response to DNA damage induced by EMS 5 mM in root meristems of *A. cepa***. Increase significant compared to control (0) at *p* ≤ 0.01 (b); decrease significant compared to EMS-challenge at *p* ≤ 0.05 (c) or 0.1 (d).

Comet assay revealed that CHX (1, 5 μM), ACD (5, 10 μM), 3AB (5, 10 μM), CAF (10, 20 μM), and APH (5, 10 μM) did not cause any observable DNA damage (Figure 8). The metabolic inhibitors at the tested concentrations did not show significant effect on the EMS-induced DNA damage. Al^3+^-conditioning (10 μM) conferred 72.33% genomic protection against the EMS-challenge. Pre-treatment with CHX (1, 5 μM), ACD (5, 10 μM), 3-AB (5, 10 μM), CAF (10, 20 μM), and APH (5, 10 μM) prior to Al^3+^-conditioning abolished the genomic protection significantly (*p* ≤ 0.05 or 0.01) to different extents (74.65, 68.63% under treatment with CHX at 1, 5 μM; 60.89, 59.15% under treatment with ACD at 5, 10 μM; 34.98, 65.56% under treatment with 3AB at 5, 10 μM; 35.11, 54.83% under treatment with CAF at 10, 20 μM; and 45.4, 36.28% under treatment with APH at 5, 10 μM).

## Discussion

### Modulation of Al^3+^-induced cell death and DNA-damage

Earlier we have reported that Al^3+^ (≥100 μM) induces DNA damage revealing the involvement of ROS generated through the Al-triggered oxidative burst (Achary and Panda, 2010; Achary et al., 2012). Cell death (Figure 3) assayed by the Evans Blue staining method indicates disintegration of the plasma membrane caused by Al^3+^ (Baker and Mock, 1994). The Evans Blue stain method has been shown to be a reliable measure of the PCD in rice seedlings subjected to Zn-stress (Chang et al., 2005). The involvement of signal transduction in ROS-mediated cell death or DNA damage so far has not been established. Eukaryotic MAPK cascades have evolved to transduce environmental and developmental signals into adaptive and programmed responses. MAPK cascades relay and amplify signals via three types of reversibly phosphorylated kinases (MAPKKK, MAPKK, and MAPK) leading to the phosphorylation of substrate proteins, whose altered activities mediate a wide array of responses, including changes in gene expression (Mishra et al., 2006; Rodriguez et al., 2010). Several reports suggest activation of MAPK by H_2_O_2_ (Varnova et al., 2002; Pitzschke and Hirt, 2006). In the present study, intact roots of *A. cepa* were pre-treated with protein kinase inhibitors including LY, PD, and 2-AP prior to challenge with Al^3+^ (800 μM). LY is an inhibitor of mammalian PI3 kinase, and its plant homologue VPS34-type is associated with the membrane trafficking (Davies et al., 2006; Vermeer et al., 2006). PD is a specific inhibitor of MAPKK (Alessi et al., 1995; Davies et al., 2006). The adenine analog 2AP, is a protein kinase inhibitor that inhibits the cyclin-dependent kinase (CDK) (Vesely et al., 1994; Huang et al., 2003). Also, 2-AP is a potent inhibitor of the double-stranded RNA (dsRNA)-activated protein kinase (PKR), a critical mediator of apoptosis (Kaufman, 1999). In the current study, LY, PD, and 2-AP significantly diminished Al-induced cell death and DNA damage (Figure 4) to different extents (LY > PD > 2-AP). Dephosphorylation of the MAPKs by specific phosphatases plays a critical role in their inactivation (Schenk and Snaar-Jagalska, 1999). SOV is an inhibitor of protein tyrosine phosphatase, whereas ENT and CAN are the inhibitors of serine/threonine protein phosphatase. ENT and CAN inhibit mammalian protein phosphatase 1 (PP1) and protein phosphatase 2A (PP2A), respectively (Li and Casida, 1992; Erdödi et al., 1995). Of these inhibitors, ENT only at 25, 50 μM and CAN at all the concentrations, 5–20 μM, significantly prevented the Al3^+^-induced cell death and DNA damage. SOV, on the contrary, was ineffective to counter the Al^3+^-induced cell death and DNA damage (Figure 5). The above inhibitors have been reported to diminish the Cu^2+^- or Zn^2+^-induced cell death in rice (*Oryza sativa*) root tissue (Chang et al., 2005; Hung et al., 2007). Furthermore, CAN is reported to counteract against the vanadate-induced MAPK activation in rice roots (Lin et al., 2005). Therefore, the findings of the present study suggested the involvement of MAPK-pathway in the Al^3+^-induced cell death and DNA damage.

### Role of signal transduction in Al^3+^-induced adaptive response to genotoxic stress

Perception of stress cues and relay of the signals that trigger adaptive responses are the key steps in plant stress tolerance (Chinnusamy et al., 2004). The MAPK pathways that regulate growth, death, differentiation, proliferation, and stress responses are highly conserved in eukaryotes including plants (Nakagami et al., 2005; Zhang et al., 2006). The ROS mediated oxidation of amino acid residues alters properties of specific cellular proteins involved in signal transduction, such as protein kinases, protein phosphatases, and transcription factors (Gordeeva et al., 2003). Studies have revealed the DNA repair machinery adaptively responds to oxidative or nitrosative stress, both *in vitro* and *in vivo* (Ramana et al., 1998). Metals including As^3+^ (Rao et al., 2011), Cd^2+^ (Jonak et al., 2004; Liu et al., 2010), Cr^6+^ (Ding et al., 2009), Cu^2+^ (Yeh et al., 2003; Jonak et al., 2004), Ni^2+^ (Chen et al., 2007), and Zn^2+^ (Lin et al., 2005) are known to activate MAPKs in a variety of plant species, which are believed to be mediated by ROS (Leonard et al., 2004; Nakagami et al., 2005). In our earlier studies, we have demonstrated that Al^3+^at low conditioning doses, comparable to oxidative agents such as praquat, rose bengal, or salicylic acid, induce adaptive response conferring genomic protection from the genotoxicity of methylmercury chloride (MMCl) or EMS (Patra et al., 2003; Achary and Panda, 2010). As revealed by the current study, the treatment protocol (Figure 2) comprising of the treatments administered at different time intervals ruled out the possibility of any direct interference of Al^3+^on the stability or activity of EMS. EMS being an alkylating agent can directly damage DNA as a result of depurination. Earlier, we have demonstrated the involvement of both ROS and Ca^2+^-channel in Al^3+^-induced adaptive response to genotoxic stress (Achary and Panda, 2010; Achary et al., 2013). Al^3+^ has been reported to activate a MAP kinase like protein in cell suspension cultures of *Coffea arabica* (Martínez-Estévez et al., 2001; Arroyo-Serralta et al., 2005). In the present study, the inhibitors of MAP kinase (Figure 6) significantly prevented the Al^3+^-induced adaptive response to genotoxic challenge of EMS in the order LY> PD > 2-AP. Treatment with 2-AP has been shown to cause cells to bypass chemical- and radiation-induced cell cycle arrest through yet unidentified mechanisms that promote cell survival (Huang et al., 2003). On the other hand, in the current study the protein phosphatase inhibitors such as SOV, ENT, and CAN were shown to be least effective in preventing the Al^3+^-induced adaptive response to genotoxic challenge of EMS (Figure 7). DNA-damaging agents are known to activate the protein kinases, triggering a protein phosphorylation cascade leading to the activation of transcription factors, which in turn alter gene expression (Yang et al., 2003b). Several genes are expressed in response to the Al-stress in *Arabidopsis* (Ezaki et al., 2004). A recent microarray analysis has revealed a total of 256 Al-responsive genes comprising 1.1% of the 24,000 genes of *Arabidopsis* genome of which 94 genes have been shown to be up-regulated and 162 have been observed to be down-regulated (Goodwin and Sutter, 2009). Furthermore, a proteomic analysis of primary tomato root tissue has identified 49 Al-stress responsive proteins (Zhou et al., 2009). Interestingly, *WAK1* (cell wall-associated receptor kinase 1) has been one of the early pathogenesis related (PR) genes that expresses WAK proteins in response to Al^3+^ in *Arabidopsis* (Sivaguru et al., 2003). Interestingly, many of the genes induced by Al^3+^ are also the common genes induced by oxidative stress, metal stress, and pathogen infection (Cruz-Ortega and Ownby, 1993; Hamel et al., 1998; Mitheofer et al., 2004). Therefore, the findings of the present study suggested involvement of the MAPK cascade-mediated signal transduction in the Al^3+^-induced adaptive response to genotoxic stress.

### Role of DNA-damage repair network in Al-induced adaptive response to genotoxic stress

To gain insight into the possible role of DNA damage response in the Al^3+^-induced adaptive response to genotoxic stress, metabolic inhibitors including CHX, ACD (Zhang et al., 2003), APH (Spadari et al., 1982), and CAF (Gascoigne et al., 1981), inhibitors of *de novo* translation, and *de novo* transcription, replication- and post-replication repair in eukaryotes, respectively, were used in the subsequent experiments of the current study (Figure 7). APH, an inhibitor of the mammalian polymerases α and δ (Wright et al., 1994), has been shown to inhibit plant polymerase α-like activity (Sala et al., 1980, 1981). CAF, a radio-protectant (Singh and Kesvan, 1991), has been reported to inhibit ATM/ATR (Sarkaria et al., 1999), delay replication fork progression and enhance homologous recombination (Johansson et al., 2006). 3-AB inhibits the synthesis of poly(ADP-ribose) by the enzyme PARP, which requires NAD as a substrate (Purnell and Whish, 1980). In the present study, CHX was found to be the most effective in removing Al^3+^-induced adaptive response to genotoxicity of EMS that was followed by 3-AB, ACD, CAF and APH (Figure 8). CHX being an inhibitor of *de novo* protein synthesis machinery has been observed to be the most effective in eliminating the adaptive response against genotoxic and DNA damage (Angelis et al., 2000; Patra et al., 2003). Studies using ACD and CHX have demonstrated that *de novo* transcription and translation are necessary for the activation of certain kinases at the levels of transcription and translation in tobacco cell suspension cultures treated with fungal elicitins (Zhang et al., 2000). The ROS-induced adaptive response (oxi-adaptive response) to H_2_O_2_, bleomycin, and methyl methanesulfonate in HeLa cells is mediated by the base excision repair (BER) of the toxic apurinic/apyrimidinic (AP) sites and DNA strand break lesions that are attributed to the activation of AP endonuclease, APE-1 (Ramana et al., 1998). 3-AB has been shown to abolish the Al^3+^-induced adaptive response to genotoxic stress imposed by MMCl in plant cells (Patra et al., 2003). Earlier, we have demonstrated that Al^3+^ at concentrations as low as 5–10 μM caused neither DNA damage nor cell cycle arrest thereby ruling out any involvement of ATR kinase, which is one of the key components of DNA damage response pathway (Rounds and Larsen, 2008; Nezames et al., 2012; Achary et al., 2013). On the contrary, the findings of the present study suggested involvement of the MAPK-DNA repair network in the Al^3+^-induced adaptive response to genotoxic stress by possibly overriding the DNA damage response pathway.

The results of the current study underscored the biphasic mode of Al^3+^ that it induced DNA damage in high concentration (800 μM), and in low concentration (10 μM) induced adaptive response, conferring genomic protection from EMS-challenge. The involvement of the same MAPK-DNA repair network in the induction of DNA damage as well as adaptive response is suggested.

## Author contributions

Whereas Brahma B. Panda is responsible to develop the research concept, design the experiments, interpretation of the results, obtain financial support, and writing the manuscript, V. Mohan M. Achary has executed the experiments and recorded the results.

### Conflict of interest statement

The authors declare that the research was conducted in the absence of any commercial or financial relationships that could be construed as a potential conflict of interest.
